# Oxidative Stress in Cardiac Tissue of Patients Undergoing Coronary Artery Bypass Graft Surgery: The Effects of Overweight and Obesity

**DOI:** 10.1155/2018/6598326

**Published:** 2018-12-17

**Authors:** Yves Gramlich, Andreas Daiber, Katja Buschmann, Matthias Oelze, Christian-Friedrich Vahl, Thomas Münzel, Ulrich Hink

**Affiliations:** ^1^Center for Cardiology/Cardiology 1, Laboratory of Molecular Cardiology of the Johannes Gutenberg University, Mainz, Germany; ^2^Department of Heart, Thoracic and Vascular Surgery of the Johannes Gutenberg University, Mainz, Germany

## Abstract

**Background:**

Obesity is one of the major cardiovascular risk factors and is associated with oxidative stress and myocardial dysfunction. We hypothesized that obesity affects cardiac function and morbidity by causing alterations in enzymatic redox patterns.

**Methods:**

Sixty-one patients undergoing coronary artery bypass grafting (CABG) were included in the study. Excessive right atrial myocardial tissue emerging from the operative connection to the extracorporeal circulation was harvested. Patients were assigned to control (*n* = 19, body mass index (BMI): <25 kg/m^2^), overweight (*n* = 25, 25 kg/m^2^ < BMI < 30 kg/m^2^), or obese (*n* = 17, BMI: >30 kg/m^2^) groups. Oxidative enzyme systems were studied directly in the cardiac muscles of patients undergoing CABG who were grouped according to BMI. Molecular biological methods and high-performance liquid chromatography were used to detect the expression and activity of oxidative enzymes and the formation of reactive oxygen species (ROS).

**Results:**

We found increased levels of ROS and increased expression of ROS-producing enzymes (i.e., p47phox, xanthine oxidase) and decreased antioxidant defense mechanisms (mitochondrial aldehyde dehydrogenase, heme oxygenase-1, and eNOS) in line with elevated inflammatory markers (vascular cell adhesion molecule-1) in the right atrial myocardial tissue and by trend also in serum (sVCAM-1 and CCL5/RANTES).

**Conclusion:**

Increasing BMI in patients undergoing CABG is related to altered myocardial redox patterns, which indicates increased oxidative stress with inadequate antioxidant compensation. These changes suggest that the myocardium of obese patients suffering from coronary artery disease is more susceptible to cardiomyopathy and possible damage by ischemia and reperfusion, for example, during cardiac surgery.

## 1. Introduction

Acute or chronic cardiovascular diseases, especially of myocardial origin, rank among the leading causes of death in Germany [1]. One of the most important risk factors for cardiovascular disease, in addition to smoking and diabetes mellitus, is obesity—a growing worldwide health problem that is associated with reduced life span [2, 3]. It is estimated that by 2020, three out of four Americans will be overweight [4]. Accordingly, obesity and disorders of the myocardium are considered important targets in therapy and research in order to lower the cardiovascular mortality and morbidity of the western population and preserve the quality of life of the elderly. An increased body mass index (BMI) is associated with an increased cardiovascular risk [5], increased left ventricular myocardial mass, and systolic and diastolic dysfunction [6–10]. While, in overweight individuals (BMI: 25–30 kg/m^2^), an increase in left ventricular myocardial mass contributes to a reasonable compensation mechanism, overcompensation is seen in obese individuals (BMI: >30 kg/m^2^) that may lead to left ventricular hypertrophy and reduced left ventricular function [11]. Accordingly, it has been shown that the cardiac muscle fibers of patients undergoing cardiac surgery show a negative correlation between the force amplitude of the contractile apparatus and BMI [12]. Although elevated BMI is known to correlate with a higher cardiovascular morbidity [13], the mechanisms responsible for the contractile dysfunction shown in overweight individuals are largely unknown. It is thought that hypoxia-induced hypertrophy, inflammation, and oxidative stress may play a prominent role in this phenomenon. Other possible triggers are adipocyte-secreted adipokines that lead to reduced NO bioavailability and increased oxidative stress [14]. Elevated BMI correlates with the extent of oxidative stress-mediated endothelial dysfunction [15]. Macrophages in adipose tissue induce inflammation and can also lead to impaired vascular contractility [16, 17]. Increased release of reactive oxygen species (ROS), for example, by NADPH oxidases and mitochondrial enzymes, results in cardiomyocyte hypertrophy, fibrosis, and metalloproteinase activation, potentially leading to progression of heart disease [18]. The majority of these findings relate to animal studies and lack of confirmation in humans. There are only few data on whether overweight patients with coronary artery disease (CAD) have significantly elevated levels of oxidative stress in cardiac tissue. Accordingly, sources of ROS production and enzymatic function pertaining to inotropic and ischemic tolerance have not been adequately elucidated, particularly with respect to normal-weight patients.

## 2. Material and Methods

### 2.1. Patient Cohort

Sixty-one patients undergoing coronary artery bypass graft surgery (CABG) were included in our study. We harvested excess right atrial myocardial tissue resulting from operative connection to the extracorporeal circulation. Patients were categorized into the following three groups: control (*n* = 19, BMI: <25 kg/m^2^), overweight (*n* = 25, 25 kg/m^2^ < BMI < 30 kg/m^2^), or obese (*n* = 17, BMI: >30 kg/m^2^) group. Patients with atrial arrhythmias or valvular heart disease and patients on dialysis were excluded from the study. Handling of all human materials and treatment of patients were in accordance with the Declaration of Helsinki. Informed consent was obtained from each patient after the study was explained to them. The local institutional ethics committee approved the study (number: 837.104.08 (6100)).

See supplemental Table S1 for the inclusion and exclusion criteria and supplemental Table S2 for the patient characteristics.

### 2.2. Isolation of Cardiac Mitochondria

Isolated mitochondria were prepared from excess right atrial myocardial tissue of the patients according to a previously published protocol for isolation of rat heart mitochondria [19]. Briefly, human myocardial tissue was glass-homogenized in HEPES buffer and subjected to cold centrifugation at 1500*g* at 4°C for 10 min and 2000*g* for 5 min. The resulting supernatant was centrifuged at 20,000*g* for 20 min, and the resulting pellet was resuspended in 1 mL of Tris buffer. The last centrifugation step was repeated, and the pellet was finally resuspended in 1 mL of Tris buffer. The protein content was determined by the Lowry method.

### 2.3. Detection of Oxidative Stress (Chemiluminescence) in Isolated Cardiac Mitochondria by High-Performance Liquid Chromatography- (HPLC-) Based Measurement of 2-Hydroxyethidium

Superoxide was measured by a modified HPLC-based method to quantify ethidium and 2-hydroxyethidium levels, as previously described [20]. Briefly, myocardial mitochondria (0.2 mg/mL) were incubated with 50 *μ*M dihydroethidium (DHE) for 30 min at 37°C in PBS buffer and stored at −80°C. Upon thawing, DHE oxidation products were extracted by the addition of 50% acetonitrile and 50% PBS, incubated (10 min), centrifuged (20 min at 20,000*g*), and filtered (30 kDa Millipore Filter, 45 min at 16,000*g*). A 50 *μ*L sample of this supernatant was subjected to HPLC analysis and measured, based on a previously described method [21, 22]. The system consisted of a control unit, two pumps, a mixer, detectors, a column oven, a degasser, and an autosampler from Jasco (Groß-Umstadt, Germany) and a C18-Nucleosil 100-3 (125 × 4) column from Macherey & Nagel (Düren, Germany). A high-pressure gradient was employed with acetonitrile, and 25 mM citrate buffer (pH 2.2) was used as the mobile phase with the following percentages of organic solvent: 0 min, 36%; 7 min, 40%; 8–12 min, 95%; and 13 min, 36%. The flow rate was 1 mL/min, and DHE was detected by its absorption at 355 nm, whereas 2-hydroxyethidium and ethidium were detected by their fluorescence (excitation: 480 nm; emission: 580 nm). The signal was normalized to the protein content of the mitochondrial preparations. Data on 2-hydroxyethidium were calibrated with respect to the superoxide formation rate by different xanthine oxidase concentrations for which the superoxide formation rate was determined by the cytochrome c assay [21].

### 2.4. Measurement of Mitochondrial Aldehyde Dehydrogenase (ALDH-2) Activity

ALDH-2 activity was assessed by HPLC using the mitochondrial fraction and 6-methoxy-2-naphthaldehyde (Monal-62) as a fluorescent substrate. A mitochondrial fraction (0.2 mg/mL) equivalent to the protein concentration (determined by “Lowry's method”) was incubated at 37°C for 30 min with Monal-62, and the reaction was terminated by the addition of benomyl (20 *μ*M)—an unspecific aldehyde dehydrogenase inhibitor. The oxidation of Monal-62 to the fluorescent naphthoic acid product [23] was traced by HPLC analysis as previously described [24].

### 2.5. Protein Expression

Protein expression and modification were assessed by a standard western and dot blot analysis using established protocols [25–27]. Isolated cardiac tissue was frozen and homogenized in liquid nitrogen. Proteins were separated by SDS-PAGE and blotted onto nitrocellulose membranes. After blocking, immunoblotting was performed with the following antibodies as described in supplemental Table S3. Detection and quantification were performed by enhanced chemiluminescence (ECL) with peroxidase-conjugated anti-rabbit/mouse (1 : 10,000, Vector Lab., Burlingame, CA) and anti-goat (1 : 5000, Santa Cruz Biotechnology, USA) secondary antibodies. Densitometric quantification of antibody-specific bands was performed with a ChemiLux Imager (CsX-1400M, Intas, Göttingen, Germany) and Gel-Pro Analyzer software (Media Cybernetics, Bethesda, MD).

### 2.6. Fluorescence-Based ROS Detection in Cardiac Tissue

ROS formation was detected by oxidative fluorescence microtopography using DHE as a fluorescent probe in cardiac cryosections (ethidium plus 2-hydroxyethdium). The method was based on a previously published protocol [27, 28]. Briefly, cardiac tissue was embedded in Tissue-Tek O.C.T.™ resin and frozen in liquid nitrogen. The embedded tissue pieces were coded anonymously and stored at −80°C until further processing. The coding allowed a blinded, independent examination of the tissue samples. Before staining with DHE (1 *μ*M) for 30 min at room temperature, frozen samples were cryosectioned. ROS detection was carried out by detecting 2-hydroxyethidium (2-HE; EOH—specific for superoxide anion radical) and ethidium (E+—unspecific oxidation product, e.g., by hydroxyl radicals or peroxidase-mediated reactions), both DHE oxidation products. ROS-derived red fluorescence was detected using a Zeiss Axiovert 40 CFL microscope, Zeiss lenses, and Axiocam MRm camera (Jena, Germany). Intensities of the DHE oxidation products' fluorescence were evaluated by densitometry.

### 2.7. Statistical Analysis

The statistical analysis was performed using SPSS (version 17, IBM). The Mann–Whitney *U* test was used to compare differences among the three study groups (control, overweight, and obese patients) [29]. Multiple significance level was set at *α* = 0.05. To control the “family-wise error rate” (FWER), we carried out Bonferroni's correction for comparison of multiple means [30]. The linear regressions were tested for statistical significance using ANOVA. All data are presented as mean ± SEM.

## 3. Results

### 3.1. Impact of Body Weight on Cardiac Oxidative Stress Levels

**Figure 1:**
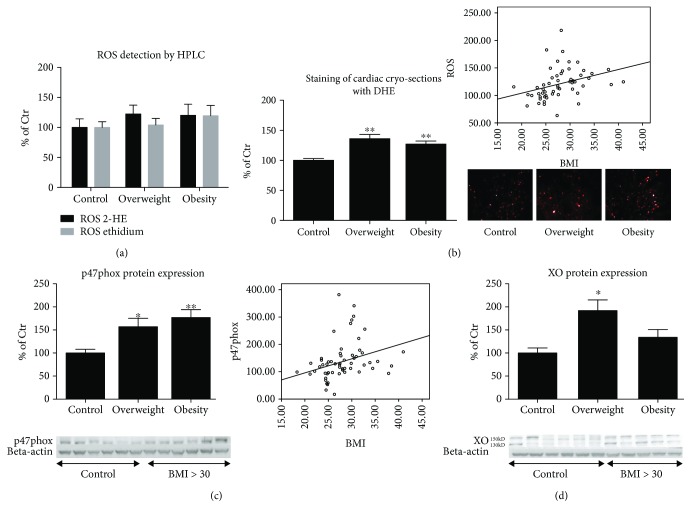
Oxidative stress parameters in cardiac tissue. (a) Determination of ROS formation in isolated cardiac mitochondria by HPLC-based quantification of 2-hydroxyethidium (2-HE) and ethidium. The values are shown as percentage of control (normal BMI). (b) Determination of ROS formation in cardiac tissue by oxidative dihydroethidium (DHE, 1 *μ*M) fluorescence microtopography in cryosections. Representative DHE-stained images are shown besides the densitometric quantification. The correlation between cardiac oxidative stress and the patient's BMI is shown along the densitometric quantification (*R* ^2^ = 0.112, *p* = 0.014). (c) Expression of the cytosolic/regulatory p47phox subunit of Nox2 isoform in cardiac tissue was determined by western blot analysis. Original blots are shown below the densitometric quantification. The signal of p47phox was normalized to the loading control—beta-actin. The correlation between BMI and the expression of p47phox is shown along the densitometric quantification (*R* ^2^ = 0.106, *p* = 0.018). (d) Determination of xanthine oxidase (XO) expression in cardiac tissue was performed by western blot analysis. Original blots are shown below the densitometric quantification. The signal of XO was normalized to the loading control—beta-actin. Control: BMI < 25 kg/m^2^; overweight: BMI 25–29 kg/m^2^; obese: BMI > 30 kg/m^2^. All data are expressed as mean ± SEM from *n* = 61 (a, b, c, d) independent measurements/patients. ^∗^ *p* < 0.05 vs. Ctr. group; ^∗∗^ *p* < 0.001 vs. Ctr. group.

To determine whether BMI directly affects the cardiac ROS levels, we evaluated ROS formation by different methods. The determination of 2-hydroxyethidium (2-HE) and ethidium (E+) by an HPLC-based assay provided evidence that the levels of ROS are somewhat elevated in isolated cardiac mitochondria of both overweight and obese patients, although not significantly (Figure 1(a)). While 2-HE is more specific for superoxide formation, ethidium is formed by a number of different oxidants (e.g., hydroxyl radicals, peroxynitrite, peroxides such as H_2_O_2_, and peroxidases). 2-HE was numerically elevated in overweight individuals by 22.5% (*p* > 0.05) and in obese patients by 20.3% (*p* > 0.05), whereas ethidium was elevated by 4.1% (*p* > 0.05) and 19.5% (*p* > 0.05), respectively. Although not statistically significant, these initial data provided a stable trend for increased cardiac mitochondrial ROS levels with respect to BMI and suggested the use of other quantitative methods for further studies. Staining of cardiac cryosections with DHE did not require isolation of mitochondria (which could already affect the ROS signals) and hence provided less specificity but better sensitivity than HPLC measurements of superoxide formation (2-HE signal). Accordingly, oxidative fluorescence microtopography provided a more general read-out of oxidative stress but with broader application than HPLC analysis [31, 32]. The fluorescence-based analysis of DHE-stained sections revealed a significant increase in the concentration of ROS in the group of overweight patients by 36% (*p* < 0.001) and obese individuals by 27% (*p* < 0.001) (Figure 1(b)). Furthermore, by using a linear regression analysis, despite a low correlation coefficient (*R* ^2^), it was observed that an increased BMI led to increased oxidative stress levels in the myocardium of all the study patients (*R* ^2^ = 0.112, *p* = 0.014) (Figure 1(b)).

These results show that obese and overweight patients with CAD have increased ROS levels in cardiac tissue, which could be related to increased production (activation of sources) or decreased detoxification (impaired antioxidant defense). In order to test for the first argument (increased ROS formation), we measured the protein expression of xanthine oxidase (XO) and p47phox, a regulatory subunit of the NADPH oxidase isoform 2 (formerly known as gp91phox-dependent phagocytic NADPH oxidase). As shown in Figures 1(c) and 1(d), the expression of p47phox and XO increased by 57% (*p* = 0.008) and by 92% (*p* = 0.005), respectively, in overweight individuals. In addition, in the obese group, an enhancement of p47phox by 77% (*p* < 0.001) and XO by 34% (*p* > 0.05) was detected. It was also found that the expression of the Nox2 major subunit—p47phox—a required subunit for activation of Nox2, is increased in overweight patients and was further augmented in obese patients (Figure 1(c)). Linear regression analysis showed that the expression of the p47phox subunit increased with elevated BMI (*R* ^2^ = 0.106, *p* = 0.018) (Figure 1(c)).

### 3.2. Impact of Body Weight on the Cardiac Nitric Oxide Pathway

**Figure 2:**
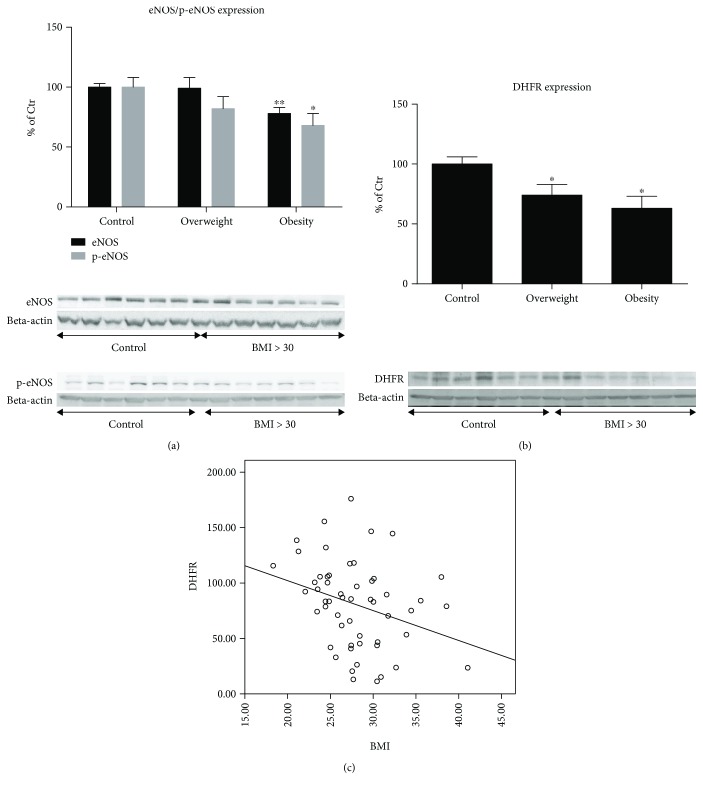
Expression of regulatory proteins of the vascular tone in cardiac tissue. (a) Determination of the expression of endothelial NO synthase (eNOS) and its activated form, pSer1177-eNOS, by western blot analysis. The values are shown as percentage of control (normal BMI). Original blots are shown below the densitometric quantification. The signals of eNOS/pSer1177-eNOS were normalized to the loading control—beta-actin. (b) Expression of dihydrofolate reductase (DHFR) was measured by western blot analysis. Original blots are shown below the densitometric quantification. The signal of DHFR was normalized to the loading control—beta-actin. (c) The correlation between the BMI and the expression of DHFR is shown along the densitometric quantification (*R* ^2^ = 0.079, *p* = 0.023). Control: BMI < 25 kg/m^2^; overweight: BMI 25–29 kg/m^2^; obese: BMI > 30 kg/m^2^. All data are expressed as mean ± SEM from *n* = 61 (a, b, c) independent measurements/patients. ^∗^ *p* < 0.05 vs. Ctr. group; ^∗∗^ *p* < 0.001 vs. Ctr. group.

In many cardiovascular disease conditions, eNOS may switch from a protective enzyme to uncoupled eNOS—the type that encourages disease progression. In its coupled state, eNOS produces the vasodilator nitric oxide that is attributed to antiaggregatory and antiatherogenic properties. Uncoupled eNOS on the other hand produces superoxide anion radicals that facilitate platelet aggregation and atherosclerosis [33–36]. Obesity induced a significant decrease in eNOS expression in cardiac tissue of 22% of the patients (*p* < 0.001) compared with the normal-weight control group, whereas the overweight patients showed no difference (Figure 2(a)), suggesting decreased NO synthesis in obese patients. Likewise, the proportion of eNOS phosphorylation at the serine 1177 (Ser1177) residue, indicative of eNOS activation, showed a decreased trend in overweight patients by 18% (*p* > 0.05) and was significantly reduced in the obese group by 32% (*p* = 0.022).

The level of tetrahydrobiopterin (BH_4_), which is the essential cofactor of eNOS, was found reduced under oxidative stress conditions due to peroxynitrite or other ROS-mediated oxidation to dihydrobiopterin (BH_2_) [37–39]. However, BH_4_ supplementation in patients with myocardial infarction, diabetes, and hypercholesterolemia improved endothelial function, a surrogate parameter of the eNOS functional state [40–42]. BH_4_ levels are largely regulated by de novo synthesis of GTP-cyclohydrolase-1 (GCH-1) or by “recycling” of BH_2_ to BH_4_ by dihydrofolate reductase (DHFR) [36]. Although no significant differences were observed among the three groups regarding the expression of GCH-1 (data not shown), the expression of DHFR decreased with increasing BMI by 26% (*p* = 0.018) in the overweight group and by 37% (*p* = 0.004) in the obese group (Figure 2(b)). This inverse correlation was supported by linear correlation analysis between DHFR expression and BMI levels (*R* ^2^ = 0.079, *p* = 0.023) (Figure 2(b)), supporting the hypothesis of reduced BH_4_ levels causing eNOS dysfunction in cardiac tissue of obese individuals.

### 3.3. Impact of Body Weight on the Cardiac Antioxidant Defense System

**Figure 3:**
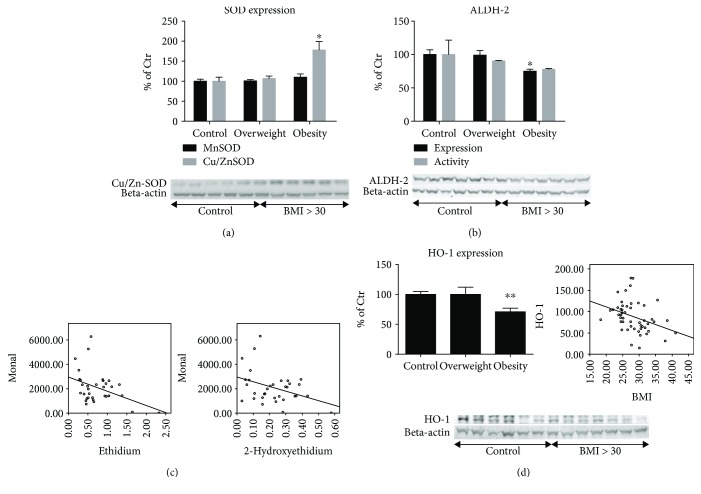
Expression of antioxidant proteins in cardiac tissue. (a) Expression of mitochondrial superoxide dismutase (Mn-SOD) and the cytosolic isoform (Cu,Zn-SOD) was determined by western blot analysis. The values are shown as percentage of control (normal BMI). Original blots are shown below the densitometric quantification. The signals of the SODs were normalized to the loading control—beta-actin. (b) Expression of the mitochondrial aldehyde dehydrogenase (ALDH-2) by western blot analysis. Original blots are shown below the densitometric quantification. The signal of the ALDH-2 was normalized to the loading control—beta-actin. ALDH-2 activity was determined by Monal-62 as a substrate and HPLC-based quantification. (c) Correlations between cardiac oxidative stress (2-HE and ethidium) and ALDH-2 activity (Monal-62) are shown. Ethidium (*R* ^2^ = 0.130, *p* = 0.022); 2-HE (*R* ^2^ = 0.111, *p* = 0.033). (d) Expression of heme oxygenase-1 (HO-1) by western blot analysis. Original blots are shown below the densitometric quantification. The signal of HO-1 was normalized to the loading control—beta-actin. The correlation between BMI and HO-1 is shown (*R* ^2^ = 0.101, *p* = 0.014). Control: BMI < 25 kg/m^2^; overweight: BMI 25–29 kg/m^2^; obese: BMI > 30 kg/m^2^. All data are expressed as mean ± SEM from *n* = 61 (a), 61 (expression)/33 (activity) (b), and 61 (c) independent measurements/patients. ^∗^ *p* < 0.05 vs. Ctr. group; ^∗∗^ *p* < 0.001 vs. Ctr. group.

As proposed above, increased oxidative stress may be a consequence of either increased ROS formation or impaired ROS detoxification. Although both mitochondrial (Mn-SOD, SOD2) and cytoplasmic (Cu,Zn-SOD, SOD1) superoxide dismutases represent important antioxidant enzymes, genetic deficiency of only the mitochondrial isoform is lethal. Importantly, administration of an encapsulated cytoplasmic isoform conferred protection against cardiac ischemia-reperfusion injury [43, 44]. Here, no differences in Mn-SOD expression were identified among groups, but a significant increase in Cu,Zn-SOD levels by 78% (*p* = 0.002) was observed in the obese group, whereas overweight individuals showed no difference (Figure 3(a)). These observations were consistent with an at least transitory compensatory response to increased superoxide production.

The mitochondrial ALDH-2 has been reported as a major cardioprotective enzyme. Its genetic deficiency increased whereas overexpression decreased infarct size and ischemic damage in animal models of myocardial infarction [45, 46]. Expression levels of ALDH-2 decreased in a BMI-dependent manner showing a significant attenuation in the obese group by 25% (*p* = 0.002) (Figure 3(b)). In a subgroup analysis, we could show that mitochondrial ALDH-2 activity decreases in a superoxide formation rate-dependent fashion resulting in a linear correlation between ALDH-2 activity and cardiac superoxide formation (*n* = 33; *R* ^2^ = 0.111, *p* = 0.033) (Figure 3(c), right graph).

Heme oxygenases are antioxidant enzymatic systems that produce the free radical scavengers biliverdin and bilirubin as well as the carbon monoxide that acts as an antiatherogenic, antiaggregatory, and vasodilatory agent and the iron-storing protein ferritin, which is responsible for transmitting the stress response. Notably, the inducible isoform—heme oxygenase-1 (HO-1)—confers high cardioprotective effects [47–50]. The expression of HO-1 was significantly decreased in obese patients by 29% (*p* < 0.001) as compared to the control group with normal BMI and showed an inverse correlation with BMI (Figure 3(d)).

### 3.4. Impact of Body Weight on Cardiac Inflammation

**Figure 4:**
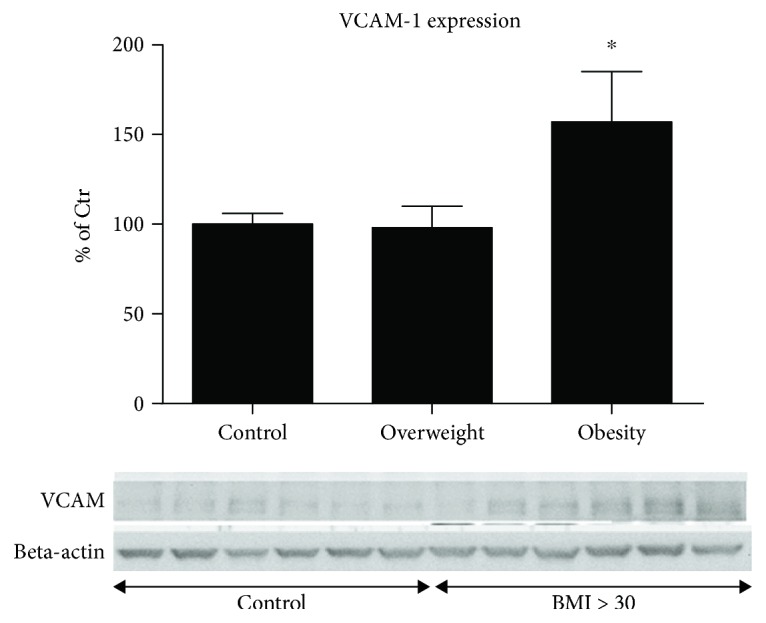
Marker of inflammation. Expression of vascular cell adhesion molecule-1 (VCAM-1) was determined by western blot analysis. The values are shown as percentage of control (normal BMI). Original blots are shown below the densitometric quantification. The signal of VCAM-1 was normalized to the loading control—beta-actin. Control: BMI < 25 kg/m^2^; overweight: BMI 25–29 kg/m^2^; obese: BMI > 30 kg/m^2^. All data are expressed as mean ± SEM from *n* = 61 independent measurements/patients. ^∗^ *p* < 0.05 vs. Ctr. group

VCAM-1 is an important vascular (mainly endothelial) adhesion protein, which initiates the first step in the adhesion of circulating immune cells, followed by infiltration of these immune cells into adjacent tissues leading to the progression of atherosclerosis and unspecific tissue damage [51]. However, adhesion and infiltration of leukocytes are also essential for the removal of infiltrating pathogens and cell debris, thereby leading to the resolution of inflammation. VCAM-1 expression can be triggered by increased oxidative stress [52, 53] but is also controlled by other cytokines. VCAM-1 expression was increased in obese patients by 63% (*p* = 0.049), whereas no difference was seen in overweight patients (Figure 4). In accordance with higher cardiac tissue VCAM-1 expression in obese patients, the markers of the proinflammatory state, sVCAM-1 and RANTES, showed an elevated trend with increasing BMI (supplemental Figure S1).

## 4. Discussion

In this study of 61 overweight CAD patients, we show an increased myocardial burden of oxidative stress and decreased expression of antioxidant and cardioprotective enzymes as well as augmented markers of inflammation. These changes suggest a role of elevated BMI in the progression of heart disease mediated by oxidative stress.

### 4.1. Oxidative Stress Promotes Pathophysiological Pathways

Reactive oxygen species (ROS) are known to cause cardiac damage at different levels: first, apoptosis can be induced by oxidative damage and altered mitochondrial permeability in a redox-dependent manner [54]. Second, in the setting of atherosclerosis, ROS may lead to plaque erosion and thrombosis [55, 56]. Third, redox-sensitive enzymes are oxidized by ROS and functionally altered [57]. In this study, we provide evidence for ROS-dependent regulation of eNOS, XO, and ALDH-2 in the myocardium of patients with increased BMI: endothelial NO synthase (eNOS) activity is altered by redox-dependent changes in BH_4_ levels [58], xanthine oxidoreductase expression by redox-dependent conversion of the xanthine dehydrogenase form to the oxidase form (reviewed in [59]), and finally ALDH-2 activity/expression by redox-dependent cysteine sulfhydryl group oxidation and altered protein degradation (reviewed in [60, 61]). Many different mechanisms (redox switches) besides BH_4_ depletion were described for oxidant-driven uncoupling of eNOS, such as S-glutathionylation in the reductase domain, adverse phosphorylation by redox-sensitive kinases, disruption of the zinc-sulfur complex in the dimer binding region, and finally dysregulated formation/degradation of the eNOS inhibitor asymmetric dimethyl arginine (ADMA) (reviewed in [59]). Fourth, the vasodilator and signal molecule nitric oxide (NO) reacts with its biological and chemical antagonist superoxide to form peroxynitrite (ONOO^−^) [62], a highly potent oxidant, also promoting the nitration and inactivation of prostacyclin synthase [63].

The decreased NO bioavailability leads to a plethora of cardiovascular complications such as increased platelet aggregation and activation, increased vascular permeability and inflammation, augmented leukocyte adhesion and infiltration into the vascular wall, progression of atherosclerosis, and plaque instability [56, 64–70]. Likewise, excess formation of peroxynitrite eliminates another important vasodilator—prostacyclin (PGI_2_) [71]. Under physiological conditions, vascular tone is regulated by endothelial cells through the production of NO and PGI_2_, decreasing intracellular calcium concentration in smooth muscle cells. NO and prostacyclin act synergistically. With increasing oxidative stress, however, prostacyclin synthase is nitrated by peroxynitrite at an essential tyrosine residue and thus inhibited [72], cyclooxygenase is activated by higher peroxide tone [73], and both mechanisms lead to the accumulation of the substrate PGH_2_, activation of the PGH_2_/thromboxane receptor, and subsequent vasoconstriction and platelet aggregation and adhesion. This was observed in diabetes-associated atherosclerosis and coronary reperfusion damage after ischemia [74, 75]. Peroxynitrite can cause multiple oxidative damage and trigger pathophysiological events such as lipid and protein oxidation [76], endothelial dysfunction, and vascular inflammation by promoting oxidation of LDL (low-density lipoprotein) to oxLDL [56, 77].

### 4.2. Sources of Oxidative Stress in Cardiovascular Disease

As sources of oxidative stress, we could identify the phagocytic NADPH oxidase isoform (Nox2 and to a lesser extent Nox1) with its regulatory subunit p47phox, XO, and potentially dysregulated endothelial NO synthase (eNOS) (Figures 1 and 2). It has already been described that the XO is significantly elevated in several conditions such as in limb ischemia [78], after major surgery [79], or in CAD [80]. In the present study, being overweight was found to be a strong trigger of increased XO expression (Figure 1(d)). Accordingly, the inhibition of XO activity improved numerous parameters that are associated with cardiac disease conditions, but this effect appears to be limited to hyperuricemic patients [81]. The role of XO in various forms of ischemic and other types of tissue and vascular injury, inflammatory diseases, and chronic heart failure seems certain, whereas current clinical trials for the therapeutic effect of XO inhibition yielded rather heterogeneous results, as reported for allopurinol, which was investigated in a decisive manner by Pacher et al. [82].

NADPH oxidases are a major source of oxidative stress in the cardiovascular system and contribute, for instance, to the pathogenesis of hypertension, atherosclerosis, myocardial infarction, myocardial hypertrophy, vascular restenosis, and arrhythmia [18, 56, 83–87]. The present study showed that the expression of p47phox increased in overweight patients by approximately 60% and in obese patients by approximately 80% (Figure 1(c)). The increase in the p47phox is consistent with the increase in global cardiac oxidative stress (Figure 1(b)), whereas specific cardiac mitochondrial oxidative stress showed a rather moderate trend of increase in dependence of BMI (Figure 1(a)). The p47phox subunit is the major Nox2 (gp91phox) regulatory subunit. Its phosphorylation is mandatory for Nox2 activation [88]. Nox2-knockout mice are protected from angiotensin II-induced hypertension and endothelial dysfunction [89], from myocardial infarction-induced damage of heart tissue [90], and from cardiac hypertrophy, cardiac fibrosis, and cardiac insufficiency [91]. Silver et al. showed an increase in p47phox in endothelial cells of obese patients with BMI > 25 kg/m^2^ and the active form of endothelial NO synthase—p-eNOS (Ser1177) [92]. In line with our findings, the Cu,Zn-SOD expression was most likely increased as a compensatory response. The authors hypothesized increased oxidative stress in obese patients. In addition, in the human myocardium, increased Nox2 expression was found in areas of myocardial infarction [93]. Therapeutic approaches to target Nox2 or its subunits are already present [56]. A key role of Nox2 in ischemic heart disease is supported by the observation that p47phox overexpression is associated with worse outcome in myocardial infarction [94].

There was a very significant decrease in eNOS expression in obese patients compared to normal-weight control subjects, by approximately 25%. Of interest, we found no difference in the group of overweight patients (Figure 2). This result was associated with a decrease in phosphorylation at serine residue 1177 (Ser1177) (activated eNOS) of about 20% and 30% in overweight and obese patients, respectively. These results suggest both a deficiency of eNOS and a decreased/dysregulated eNOS activity (p-eNOS Ser1177) in the cardiac tissue of these patients, which may lead to not only a loss of the protective properties but also an uncoupling of eNOS and subsequent superoxide production. One possible reason for the pathogenesis of cardiomyopathy in metabolic syndrome may be based on the deficiency of functional eNOS, which has already been demonstrated in animal models [95, 96]. The excessive formation of peroxynitrite and the overall increased oxidative stress in cardiac tissue of overweight and obese patients have been demonstrated in this work, which can be interpreted as a transformation of the protective physiological properties of NO to pathophysiological properties mediated by peroxynitrite [36, 97, 98]. Among other pathways, oxidative depletion of BH_4_ was demonstrated as an important trigger of eNOS dysfunction. Of note, DHFR is responsible for “recycling” BH_2_ to BH_4_ [99, 100]. Our results indicate, for the first time to our knowledge, a deficiency of DHFR in the cardiac tissue of patients with increasing BMI (Figure 2(c)) and suggest an impaired cardiac BH_4_ metabolism in obesity. In a previous study, Denk et al. demonstrated decreased contractility of the cardiac muscle of obese patients [12], which may be explained by the impaired NO system (Figure 2), a hypothesis that is also supported by studies in animal models [101, 102].

### 4.3. Impact of BMI on Cardiac Antioxidant Defense Systems

An increase in the aforementioned prooxidative processes may be attributed to not only activated sources of ROS formation but also impaired antioxidant defense systems such as SOD, ALDH-2, and inducible heme oxygenase (HO-1). Although no change was observed in mitochondrial Mn-SOD (SOD-2) expression, the cytoplasmic Cu,Zn-SOD (SOD-1) was increased in obese patients (Figure 3(a)), which most likely reflects a compensatory mechanism [103, 104] in a more profound disease state. Upregulation of SOD-1 protects against endothelial dysfunction and ischemic damage [24, 105]. One of the main findings of this work is the BMI-dependent decreased expression and ROS-dependent inhibition of ALDH-2 activity in the cardiac tissue of obese individuals (Figure 3(b)). Several animal and human studies have shown that ALDH-2 is regulated/inhibited in conditions of increased oxidative stress [45, 106]. In the present study, to our best knowledge, we show for the first time that the level of ALDH-2 inhibition is directly correlated with the superoxide formation rate in the human myocardium (Figure 3(c)) and that its expression is negatively influenced by obesity (Figure 3(b)). In recent publications, a larger ischemic heart damage and further reduced inotropy were demonstrated in animals with reduced ALDH-2 expression [94, 99, 100]. In addition, we demonstrated that eNOS expression was reduced, which also contributes to reduced inotropy [107, 108]. Taken together, a lower antioxidant potency, increased myocardial vulnerability, especially to aldehyde stress, and a lower overall cardioprotection by the ALDH-2 enzyme in obese patients must be postulated [109, 110]. The lower expression of HO-1 in the cardiac tissue of obese patients is a further indicator for reduced antioxidant protection in the myocardium of obese patients with CAD (Figure 3(d)). A possible mechanism may be a lack of induction of HO-1 by NO or adiponectin.

### 4.4. Impact of BMI on the Cardiac Expression of VCAM-1 Adhesion Molecule as a Marker of Local Inflammation

The VCAM-1 protein is an endothelial adhesion molecule, which triggers adhesion of lymphocytes, monocytes, eosinophils, and basophils to the endothelial cell layer. Its expression is triggered by cytokines, and it is considered a marker of inflammation. It is assumed that VCAM-1 plays a role in the pathogenesis of cardiovascular disease, especially atherosclerosis and rheumatoid arthritis [111]. Western blotting analysis revealed an increase in VCAM-1 expression by 63% (*p* = 0.049) in the group of obese patients; however, there were no differences seen between overweight and control group patients. These observations suggest local inflammation in obesity, supported by studies that showed a positive correlation of visceral fat content with higher levels of procoagulant plasminogen activator inhibitor 1 (PAI-1) that links adipokines to “low-grade inflammation.”

## 5. Conclusion

**Figure 5:**
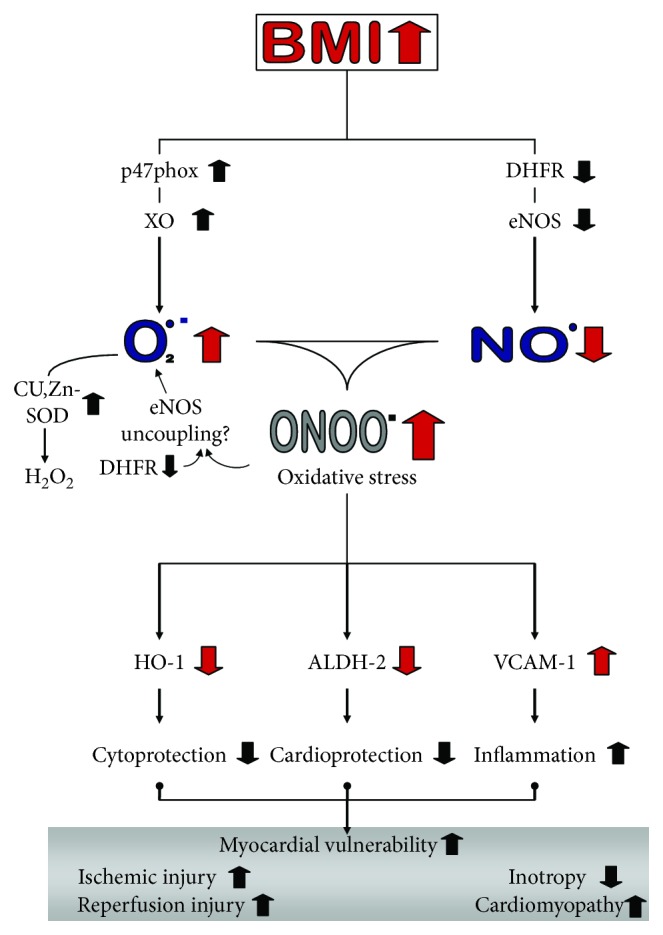
Schematic summarizing the major findings and providing a hypothesis on the major pathways that lead to cardiac and vascular dysfunction in obese patients.

In summary, the myocardium of patients with CAD with increasing BMI shows increased oxidative stress and enzymatic alterations suggestive of inadequate antioxidant defense (Figure 5). We therefore suggest that the myocardium of overweight patients is more susceptible to damage caused by ischemia and reperfusion during cardiac surgery or acute coronary syndromes and to the development of cardiomyopathies.

## Data Availability

Since all the data used for this article are deposited in the archive of the IZKS of the University Medical Center Mainz together with the patient personal information, a public access cannot be granted. The involved study physicians have access to the data via SPSS within the campus network and can provide raw data without patients' personal information on reasonable request.

## Supplementary Materials

Supplementary Materials Table S1: patient inclusion and exclusion criteria. Table S2: patient characteristics. Table S3: details on used antibodies. Figure S1: ELISA for sVCAM-1 and RANTES.
