# Supplementary material for: Oxidative Stress in Cardiac Tissue of Patients Undergoing Coronary Artery Bypass Graft Surgery: The Effects of Overweight and Obesity
Source: Oxid Med Cell Longev. 2018 Dec 17;2018:6598326. doi: 10.1155/2018/6598326 (PMC6311809; doi:10.1155/2018/6598326)
Supplement: Supplementary Materials — Table S1: patient inclusion and exclusion criteria. Table S2: patient characteristics. Table S3: details on used antibodies. Figure S1: ELISA for sVCAM-1 and RANTES. [file 6598326.f1.pdf]

Online data supplement

Oxidative stress in patients undergoing coronary artery bypass graft surgery: the effects of overweight and obesity

Yves Gramlich[1], Andreas Daiber[1], Katja Buschmann[2], Matthias Oelze[1], Christian-Friedrich Vahl[2], Thomas Münzel[1] and Ulrich Hink[1]

[1] Center for Cardiology, Cardiology 1, University Medical Center Mainz, Germany. [2] Clinic of Thoracic
and Cardiovascular Surgery, University Medical Center Mainz, Germany

Supplemental Table S1. Patient inclusion and exclusion criteria.

Inclusion criteria
• First-time bypass surgery<br>• Consent of the patient for surgery, as well as for research projects such as the ethics application (IRB approval 837.104.08 (6101))<br>• Personal signature on the study information sheet
**Exclusion criteria**
• Rejection by patient<br>• Mentally ill patients or patients who have had a guardian care<br>• Rejection by surgeon<br>• "off-pump" operations<br>• Bypass Revision (so-called. RE-ACB)<br>• Requiring valvular surgery<br>• Pre-existing conditions of the rheumatic diseases<br>• Condition for any transplant<br>• Infectious diseases (HIV, Hepatitis, O.A.)<br>• Emergency surgery

Supplemental Table S2. Patient characteristics.

		BMI < 25 kg/m²	BMI 25-30 kg/m²	BMI > 30 kg/m²	Total
Patients	n	19	25	17	61
	%	31,10%	41,00%	27,90%	100,00%
Diebetes Mellitus	n	8	3	6	17
(IDDM/NIDDM)	%	42,10%	12,00%	37,50%	27,87%
Gender					
Male	n	16	18	8	42
	%	84,20%	72,00%	47,10%	68,90%
Female	n	3	7	9	19
	%	15,80%	28,00%	52,90%	31,10%
Age	Min.	53	46	50	46
	Max.	82	81	80	82
	Average	66,89	68,32	62,06	66,13
	SEM	2,06	1,93	1,99	1,19
Medication					
ACE/AT1	n	4	4	2	10
	%	21%	16%	12%	16%
Statin	n	13	14	10	37
	%	68%	56%	59%	61%
Betablocker	n	11	12	13	36
	%	58%	48%	76%	59%
Nitro	n	1	2	3	6
	%	5%	8%	18%	10%
Spironolacton	n	1	1	0	2
	%	5%	4%	0%	3%
Clopidogrel	n	2	4	6	12
	%	11%	16%	35%	20%
ASS100	n	16	18	11	45
	%	84%	72%	65%	74%
Hypertonie	n	10	11	12	33
	%	53%	44%	71%	54%
Stenosis					
LAD	n	8	11	7	26
	%	42%	44%	41%	43%
RCX	n	10	10	8	28
	%	53%	40%	47%	46%
RCA	n	9	10	6	25
	%	47%	40%	35%	41%
MS	n	2	5	1	8
	%	11%	20%	6%	13%
Lipid					
HDL	Average	37,05	43,59	31,17	37,27
	Min	31,35	22,88	22,96	25,73
	Max	41,80	123,88	53,64	73,11
LDL	Average	90,88	117,03	112,52	106,81
	Min	32,31	190,00	92,43	104,91
	Max	179,23	56,21	136,56	124,00
TG	Average	90,43	129,09	185,25	134,92
	Min	41,00	68,00	118,00	75,67
	Max	133,00	245,00	239,00	205,67
Cholesterin	Average	136,71	155,64	154,00	148,78
	Min	73,00	88,00	127,00	96,00
	Max	255,00	224,00	184,00	221,00
LVEF	Average	55,00	60,00	72,00	62,33
	Min	46,00	32,00	60,00	46,00
	Max	64,00	83,00	83,00	76,67

Supplemental Table S3. Details on used antibodies.

	kDa	Delution
Loading-Control:		
ß-Actin (Sigma)	42	1:2500
NO synthase:		
eNOS (BD Bioscience)	140	1:1000
p-eNOS_Ser1177 (BD Bioscience)	140	1:1000
GCH-1 (SantaCruz)	~26	1:100
DHFR (Abnova)	21	1:500
NADPH oxidase:		
Nox1 (=Mox1, SantaCruz)	~60	1:100
Nox2 (=gp91Phox; BD Biosience)	58	1:500
p47phox (BD Biosience)	47	1:250
Antioxidant enzymes:		
HO-1 (StressGen)	31	1:250
Mn-SOD (SOD-2; Upstate)	24	1 µg/ml
Cu/Zn-SOD (SOD-1; Upstate)	16	1:1000
Others:		
ALDH-2 (Kwok K. Ho, Indiana, USA)	52	1:2500
Xanthine oxidase (LabVision)	150	1 :2000
VCAM-1	110	1:250

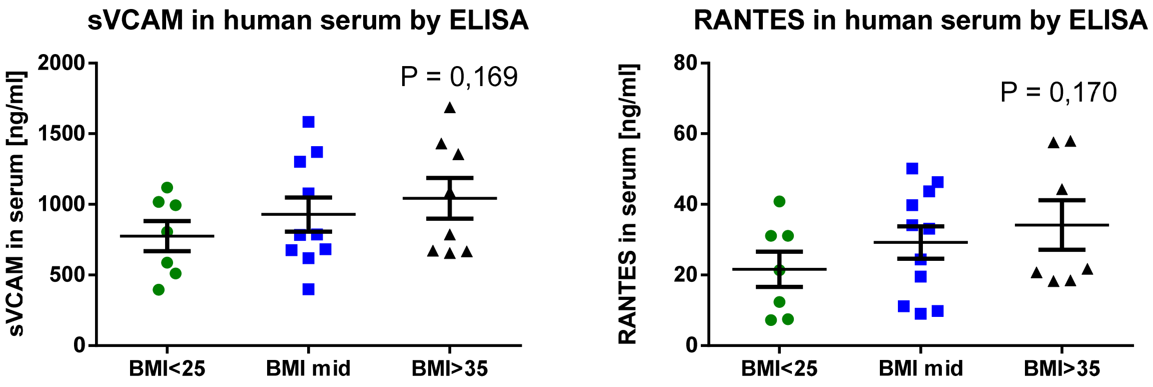

Supplemental Figure S1. ELISA for sVCAM-1 and RANTES.

ELISA of sVCAM-1 and RANTES were performed in a subgroup of 26 patients. In accordance with higher cardiac tissue VCAM-1 expression in obese patients, the markers of pro-inflammatory state, sVCAM-1 and RANTES, were elevated by trend with increasing BMI. Quantikine ELISA Immunoassays were performed according to the manufacturer‘s instructions and were obtained from R&D Systems (human CCL5/RANTES and sVCAM-1).
